# Can you be a manual therapist without using your hands?

**DOI:** 10.1186/s12998-022-00457-x

**Published:** 2022-11-14

**Authors:** Bruno T. Saragiotto, Louise F. Sandal, Jan Hartvigsen

**Affiliations:** 1 Masters and Doctoral Programs in Physical Therapy, Universidade Cidade de São Paulo, R. Cesário Galero, 448/475 - Tatuapé, São Paulo, SP 03071-000 Brazil; 2 Discipline of Physiotherapy, Graduate School of Health, University of Technology Sydney, Sydney, Australia; 3 Center for Muscle and Joint Health, Department of Sports Science and Clinical Biomechanics, University of Southern Denmark, Odense M, Denmark; 4 Chiropractic Knowledge Hub, Odense M, Denmark

**Keywords:** Chronic pain, Telehealth, Telerehabilitation, Online, Musculoskeletal pain, Digital health

## Abstract

**Background:**

To align with current best practices, manual therapists have refined their treatment options to include exercise and pain education for people with chronic musculoskeletal pain. In this commentary, we suggest that manual therapists should also add telehealth to their toolbox. Thus, we aim to discuss the use of telehealth by manual therapists caring for patients with musculoskeletal disorders.

**Main body:**

Telehealth can be delivered to the patient in different modes, such as real-time clinical contact or asynchronously. Platforms vary from websites and smartphone apps to virtual reality systems. Telehealth may be an effective approach, especially for improving pain and function in people with musculoskeletal pain, and it has the potential to reduce the individual and socioeconomic burden of musculoskeletal conditions. However, the certainty of evidence reported in systematic reviews is often low. Factors such as convenience, flexibility, undivided attention from the clinician, user-friendly platforms, goal setting, and use of evidence-based information are all enablers for telehealth use and improving patients’ knowledge, self-efficacy, and self-management. Barriers to widening the use of telehealth in musculoskeletal care include the reliability of technology, data privacy issues, difficult to build therapeutic alliance, one-size-fits-all approaches, digital health literacy, and payment models.

**Conclusion:**

We suggest that practitioners of manual medicine make telehealth part of their clinical toolbox where it makes sense and where there is evidence that it is beneficial for people who seek their care.

## Background

Telehealth can be defined as the use of electronic information and telecommunications technologies to support long-distance clinical health care [1]. Telehealth is not a new approach; it has been around for half a century. One of the first reports of the use of telehealth, from the 1970s, used black and white television systems and telephone consultations to deliver care to remote, medically underserved areas [2, 3]. The integration between audio and video was only possible via satellite, microwave, or cable. It required the bandwidth of a thousand telephones, which was very expensive and unfeasible at that time. Today, advances in technology and telecommunication have enabled an exponential increase in telehealth initiatives. These advances include videoconferencing, store-and-forward information, patient monitoring (wearables, devices, self-report), streaming, and communication, and can assist with diagnosis, treatment, or prevention of a condition.

Telehealth has the potential to reach every population in the world. In high-income countries, more than two-thirds of the population has internet access. Low- and middle-income countries report much lower rates, but access is increasing substantially [4], and soon disadvantaged populations may have better access to the internet than to in-person quality health services [5]. Despite its great potential, telehealth is still in its infancy and faces many implementation barriers, especially from clinicians [6]. Manual therapists such as chiropractors define themselves by the physical clinical encounter with a patient and primarily rely on hands-on approaches. Over the years, however, most have added other strategies such as exercise and pain education to their toolbox, evolving to more complete and guideline-based care offered to their patients [7]. We suggest it is time they also add telehealth to that toolbox. This commentary discusses the use of telehealth by manual therapists.

## How telehealth has been used in musculoskeletal practice

Telehealth can be delivered to the patient in different modes, and interventions are often multimodal. One characteristic is whether the telehealth intervention includes a clinician with whom the patient can communicate. Clinician contact can be in real time, where the patient directly sees and talks with the clinician, or it can be asynchronous, where the communication is direct with the patient, but responses may be delayed and delivered via email, voice messages, or via images, videos, or other messages. Interventions not involving clinicians directly can be characterized by the platform, i.e., website, app, virtual reality, or audio/podcasts. The level of interaction through the platform can also vary as content and tools can be static, active, or interactive, and the level of personalization and use of methodology to tailor or personalize can vary.

### Effectiveness of telehealth for musculoskeletal care

Previous studies have tested many different telehealth approaches. Most telehealth modalities benefit people with musculoskeletal pain by improving pain and function and thus show potential to reduce the individual and societal burden imposed by musculoskeletal conditions. However, systematic reviews report the certainty of this evidence as low [8–15]. One important limitation is that most systematic reviews pooled results across a range of interventions (e.g., physical and psychological therapies, education, multimodal), so there is high clinical heterogeneity. Consequently, there is uncertainty around the best approach regarding telehealth delivery, and it is still uncertain what is the best modality of telehealth for specific conditions and therapies in terms of effectiveness, cost and adherence. Nonetheless, patients have reported excellent satisfaction and acceptance of telehealth initiatives [16, 17]. A 2022 systematic review and meta-analysis of the effectiveness of digital health interventions for musculoskeletal pain did not identify enough studies evaluating the cost-effectiveness of these interventions, and this will be a priority for future trials[18]. Nevertheless, a recent trial on telehealth-delivered exercise and dietary weight loss programs for knee osteoarthritis reported that telehealth are likely to be cost-effective ($45,500 per QALY)[19].

### Examples of telehealth strategies in musculoskeletal care

As an example of a successful telehealth intervention, Bennell and colleagues tested the effectiveness of a real-time internet-delivered exercise and pain-coping skills training intervention delivered online in people with chronic knee pain [20]. The intervention comprised three different elements; 1) educational material delivered via a website, 2) an interactive automated Pain-coping skills training program (pain COACH) and finally, 7 Skype sessions over 12 weeks, with a duration of 30–45 min, versus having access to the website only. The trial showed that participants in the interactive arm reduced pain (mean difference and 95% CI: 1.6 points, 0.9 to 2.3 points, NRS 0–10 scale) and improved function at 3 months (mean difference and 95% CI: 9.3 points, 5.9 to 12.7, WOMAC 0–68 scale) and the effect was sustained at 9 months. Interviews with physiotherapists revealed that they were initially uncomfortable with being unable to touch their patients. However, they also reported that the project stimulated self-reflection on their delivery of care and a stronger focus on the most important and effective treatment for the patient’s knee osteoarthritis, namely exercise, education, and self-management skills, rather than hands-on examination and clinical findings [21].

Telehealth interventions utilizing no direct interaction between patients and clinicians are also becoming frequent, especially when delivered via smartphone applications [22, 23]. Using low back pain as an example, many of the available mHealth apps have not been comprehensively tested with appropriate trial designs [24]. However, evidence-based interventions underpinned by research are growing rapidly [23, 25]. The selfBACK app is an example of a digital decision system that supports patients with low back pain in their self-management [26]. In selfBACK, the patient continuously provides information about their symptoms and reports of exercise and physical activity via the app, which the digital system then uses to provide individually tailored and evidence-based content to the patient using artificial intelligence and machine learning [26]. Low back pain patients who used the selfBACK system as an adjunct to usual care had reduced pain-related disability at 3 months, with 52% in the intervention group achieving a clinically significant improvement versus 39% in the control group. While the average improvement in pain-related disability was moderate in both the intervention (3.7 (SD: 4.5) RMDQ points) and the control groups (3.0 (SD: 4.5) RMDQ points), the between-group difference was small and of uncertain clinical significance, but sustained at 9 months and consistently seen in secondary outcomes [27]. Importantly, the selfBACK intervention was an adjunct to usual care as a tool to support self-management, not as a substitution for clinical care.

Another example is the *Reabilitador* program, an asynchronous system of telerehabilitation based on exercise, education, and coaching designed for people with chronic musculoskeletal pain [28]. The program utilizes a website with a tailored patient area secured by login and password. Each week the program is updated according to the patient progress. Patients have reported high levels of satisfaction and acceptability and found the content appropriate for their condition [29]. The program was associated with a reduction in pain intensity after 8 weeks, but no significant changes in function were seen [29].

## Barriers and enablers to broader use of telehealth in musculoskeletal care

According to the literature, one of the main barriers to using telehealth is the reliability of technologies, with users emphasizing that trustworthy connections and audio-visual quality are important considerations [30, 31]. Clinicians should understand the technology available and make sure they can provide a reliable experience for patients while also planning for backup contingencies in case of technical problems [32]. Also, feeling comfortable with technologies, i.e., using standard and familiar platforms, can enhance adherence and uptake [33]. Some patients have also expressed concerns regarding data- and privacy protection [31, 34]. Therefore, health systems and clinicians must secure privacy and adhere to data protection rules and regulations such as the European General Data Protection Regulation (GDPR) or the Brazilian General Law on Protection of Personal Data (LGPD). These concerns can be addressed with end-to-end encryption and authentication to create a secure communication environment.

The clinician-patient relationship is different in the digital environment and may be perceived as a barrier to many telehealth solutions. The lack of physical presence, no touch, and lack of non-verbal communication are contributing factors to lower engagement in telehealth interventions [31]. Surprisingly, participants in a telehealth trial for knee osteoarthritis perceived the telehealth intervention to be more personal rather than less compared to physical consultations [21], a finding confirmed by subsequent studies [30]. In addition, just like for a clinical encounter, one size does not fit all, so a person-based approach to the tailoring of telehealth interventions that promote autonomy, competence, and adherence is preferred, whereas telehealth interventions that are impersonal, with limited flexibility and less interactive are seen as less engaging and not likely to meet the patient's needs [31].

A strong barrier to delivering telehealth interventions is digital health literacy, which is the ability to seek, find, understand, and appraise health information from electronic sources and apply this knowledge to a health problem [35]. Digital health literacy is a dynamic concept that is influenced by many factors, such as the patient underlying health condition, educational background, health status, motivation for seeking information, and the technologies used [36, 37]. Low digital health literacy can be associated with poor engagement in telehealth interventions, feeling of frustration and dependence on others [31]. Further, a person that is generally low engaged with digital technologies is likely to be less actively participating in their health decisions, have less control of their problem and be less able to keep up with technological advances [38]. By considering these digital skills, a clinician can open new opportunities to better engage and interact with patients during telehealth encounters.

The strongest enabler of telehealth is its convenience and flexibility. Telehealth usually requires less or no travel time and less or no time off work compared to in-person care because the patient can access telehealth services in the convenience of their own home and not in a clinical environment [30, 31]. Receiving exclusive attention from the clinician in videoconferencing and easy-to-use platforms are also positive components for improving engagement in telehealth interventions [31]. Patients also appreciate the ability to track their progress electronically, set goals, and receive continuous feedback. The use of evidence-based information can also be seen as a facilitator since it improves knowledge, self-efficacy and self-management [31]. There is also emerging evidence that telehealth can reduce costs; however, savings may not apply to healthcare consultations but rather be related to time and travel and time off work or out of daily duties while travelling [39]. Table 1 presents common challenges in telehealth with potential solutions.

**Table 1:** Challenges and potential solutions in telehealth for musculoskeletal care that clinicians should be aware of

Challenge	Solution
Reliability of technology	Technical issues will happen eventually. Plan for access to alternative, simpler technologies such as telephone or asynchronous materials in case of technical difficulties
Data privacy and sharing	Comply with data protection rules in your jurisdiction. Consider using end-to-end encryption and authentication
Clinician-patient relationship	Use telehealth in combination with physical consultations. Practice communication skills online with colleagues and friends. Avoid using technical language and information overload. Use a patient-centered approach and include patient preferences and circumstances in the structure of the sessions (e.g., work together with the patient to identify barriers and help them implement potential solutions)
Irrelevant content	Rely on evidence-based information and make content interactive and engaging. Encourage patients actively participate during sessions. Consider engaging online features such as quizzes, videos, and smartphone apps and set goals
Digital health literacy	Prepare a brief guide on how to use the technology in a booklet or video format and provide to patients a priori. Allow more time before the first sessions so the patient can get used to the technology. Offer help and assistance before and after the telehealth delivery
Clinician resistance	Explain that telehealth can be a supplement to clinical care that improves patient engagement in their care and is convenient. Communicate evidence for benefits and effectiveness
Payment	Work with healthcare administrators and third-party payers to develop sustainable business models for telehealth delivery

## Conclusion

Access to telehealth services may be suboptimal, especially in low- and middle-income countries, but every day more than 600,000 people access the internet for the first time, and there are now more than 7 billion users of mobile phones—about equal to the number of people on the planet [4, 40]. Access to online information, therefore, is everywhere and will continue to grow, making it the most powerful vehicle to spread information, including health information and potentially healthcare for individuals. We suggest that practitioners of manual medicine make telehealth part of their clinical toolbox where it makes sense and where there is evidence that it is beneficial for people who seek their care.

## Data Availability

Not applicable.
